# Validation of a novel 3-dimensional classification for degenerative arthritis of the shoulder

**DOI:** 10.1007/s00402-023-04890-2

**Published:** 2023-06-12

**Authors:** Benjamin D. Kleim, Sebastian Lappen, Pavel Kadantsev, Hannes Degenhardt, Lorenz Fritsch, Sebastian Siebenlist, Maximilian Hinz

**Affiliations:** Department of Sports Orthopaedics, Technical University of Munich, Ismaninger Str 22, 81675 Munich, Germany

**Keywords:** Classification, Validation, Interobserver, Intraobserver, Reproducibility, Shoulder arthritis, Osteoarthritis, Cuff tear arthropathy

## Abstract

**Introduction:**

A novel three-dimensional classification to comprehensively describe degenerative arthritis of the shoulder (DAS) was recently published by our group. The purpose of the present work was to investigate intra- and interobserver agreement as well as validity for the three-dimensional classification.

**Materials and methods:**

Preoperative computed tomography (CT) scans of 100 patients who had undergone shoulder arthroplasty for DAS were randomly selected. Four observers independently classified the CT scans twice, with an interval of 4 weeks, after prior three-dimensional reconstruction of the scapula plane using a clinical image viewing software. Shoulders were classified according to biplanar humeroscapular alignment as posterior, centered or anterior (> 20% posterior, centered, > 5% anterior subluxation of humeral head radius) and superior, centered or inferior (> 5% inferior, centered, > 20% superior subluxation of humeral head radius). Glenoid erosion was graded 1–3. Gold-standard values based on precise measurements from the primary study were used for validity calculations. Observers timed themselves during classification. Cohen’s weighted κ was employed for agreement analysis.

**Results:**

Intraobserver agreement was substantial (*κ* = 0.71). Interobserver agreement was moderate with a mean κ of 0.46. When the additional descriptors extra-posterior and extra-superior were included, agreement did not change substantially (*κ* = 0.44). When agreement for biplanar alignment alone was analyzed, κ was 0.55. The validity analysis reached moderate agreement (*κ* = 0.48). Observers took on average 2 min and 47 s (range 45 s to 4 min and 1 s) per CT for classification.

**Conclusions:**

The three-dimensional classification for DAS is valid. Despite being more comprehensive, the classification shows intra- and interobserver agreement comparable to previously established classifications for DAS. Being quantifiable, this has potential for improvement with automated algorithm-based software analysis in the future. The classification can be applied in under 5 min and thus can be used in clinical practice.

## Introduction

Degenerative arthritis of the shoulder (DAS) has traditionally been classified as either primary osteoarthritis (OA) or cuff tear arthropathy (CTA). Anteroposterior subluxation and glenoid morphology in the axial plane for OA is to date commonly described using the modified Walch classification [1, 2]. Superoinferior subluxation and glenoid erosion for CTA has commonly been described in the coronal plane (anteroposterior X-ray) according to the Hamada or Visotsky-Seebauer and Favard classifications, respectively [3–5]. These classifications were originally developed for describing X-ray findings and in the case of the modified Walch classification were later adapted for axial computed tomography (CT).

In recent years, however, several authors described biplanar eccentricity in glenoid erosion patterns [6–10]. OA patients often develop rotator cuff insufficiency as the disease progresses, or the disease may initially be influenced by rotator cuff degeneration [11–13]. Especially in early disease, axial plane imaging of a lying patient is more sensitive for superior subluxation than standing X-ray [14].

Therefore, a new three-dimensional (3D) classification, which categorizes anteroposterior (A-P) and superoinferior (S-I) alignment with erosion for DAS, was developed and recently published [15].

Whilst this novel classification allows for a more comprehensive description of DAS, it has not yet been validated. The higher number of categories compared to previous classifications may reduce reproducibility [1, 3, 4]. However, alignment is quantifiable in this novel classification, making this less subjective and possibly ameliorating the effect of more categories on reproducibility. Furthermore, the complex nature of the 3D classification and need for CT reconstruction could make it too time consuming for clinical practice, which requires investigation.

The purpose of this study was to validate the 3D classification for patients with DAS. The hypothesis was that validity as well as interobserver reliability would be moderate and, therefore, comparable to values for previous classifications from the literature (Table 1) [16–20].

**Table 1:** Summary of a PubMed search for studies investigating intraobserver and interobserver agreement for the Walch or modified Walch classification published in the last 5 years and Hamada, Visotsky-Seebauer and Favard classifications published in the last 15 years

Study	Classifications investigated	Imaging modality	Intraobserver agreement	Interobserver agreement
Ricchetti et al. 2021 [16]	Modified Walch	CT using 3D image viewer	Moderate to substantial (*κ* = 0.51–0.61)	Moderate (*κ* = 0.43)
Hopkins et al. 2021 [18]	Walch	CT and MRI	CT: Substantial (*κ* = 0.71) MRI: Substantial (*κ* = 0.71)	CT: Fair (*κ* = 0.29) MRI: Fair (*κ* = 0.34)
Shukla et al. 2019 [17]	Modified Walch	X-ray and CT	CT: Substantial (*κ* = 0.73) X-ray: Substantial (*κ* = 0.73)	CT: Moderate (*κ* = 0.52) X-ray: Moderate (*κ* = 0.55)
Kappe et al. 2011 [19]	Hamada, Visotsky-Seebauer and Favard	X-ray	Hamada: Substantial (*κ* = 0.75) V-S: Substantial (*κ* = 0.73) Favard: Substantial (*κ* = 0.76)	Hamada: Moderate (*κ* = 0.41) V-S: Moderate (κ = 0.55) Favard: Fair (*κ* = 0.31)
Iannotti et al. 2010 [20]	Visotsky-Seebauer, Favard and Hamada	X-ray	V-S: Substantial (*κ* = 0.69) Hamada: Almost perfect (*κ* = 0.87) Favard: Moderate (*κ* = 0.59)	V-S: Fair (*κ* = 0.39) Hamada: Moderate (*κ* = 0.42) Favard: None to slight (*κ* = 0.13)

*V-S* Visotsky-Seebauer; *κ* kappa; 3D 3-dimensional; *CT* computed tomography; *MRI* magnetic resonance imaging

## Materials and methods

### Patient population

For this validation study, a previously investigated cohort of patients with DAS who underwent primary shoulder arthroplasty (total shoulder arthroplasty, hemiarthroplasty or reverse shoulder arthroplasty) at the Department of Orthopaedic Sports Medicine of the University Hospital Rechts der Isar in Munich between 2009 and 2020 were identified [15]. 299 shoulder arthroplasty cases were performed in this period for DAS. 135 of these had preoperative CT scans taken according to a standardized in-house protocol (pitch, 0.39; slice thickness, 0.9 mm; tube voltage, 120 kV; tube current, 82 mA [range, 50–115 mA]) available for analysis. CT scans had been taken no more than 6 months prior to surgery. Five of these patients were excluded from analysis: in two cases, the scapula was not adequately exposed, one due to movement artefact and two due to severe erosion, due to which landmarks for measurement could not be reliably placed. Of the remaining 130 CTs, 100 were selected at random.

Demographically the mean patient age was 70 years (range 38–88 years) and 49 were male (49%). 59 patients had a preoperative diagnosis of CTA and 41 of OA, as documented in the operative reports.

### CT classification

**Fig. 1:**
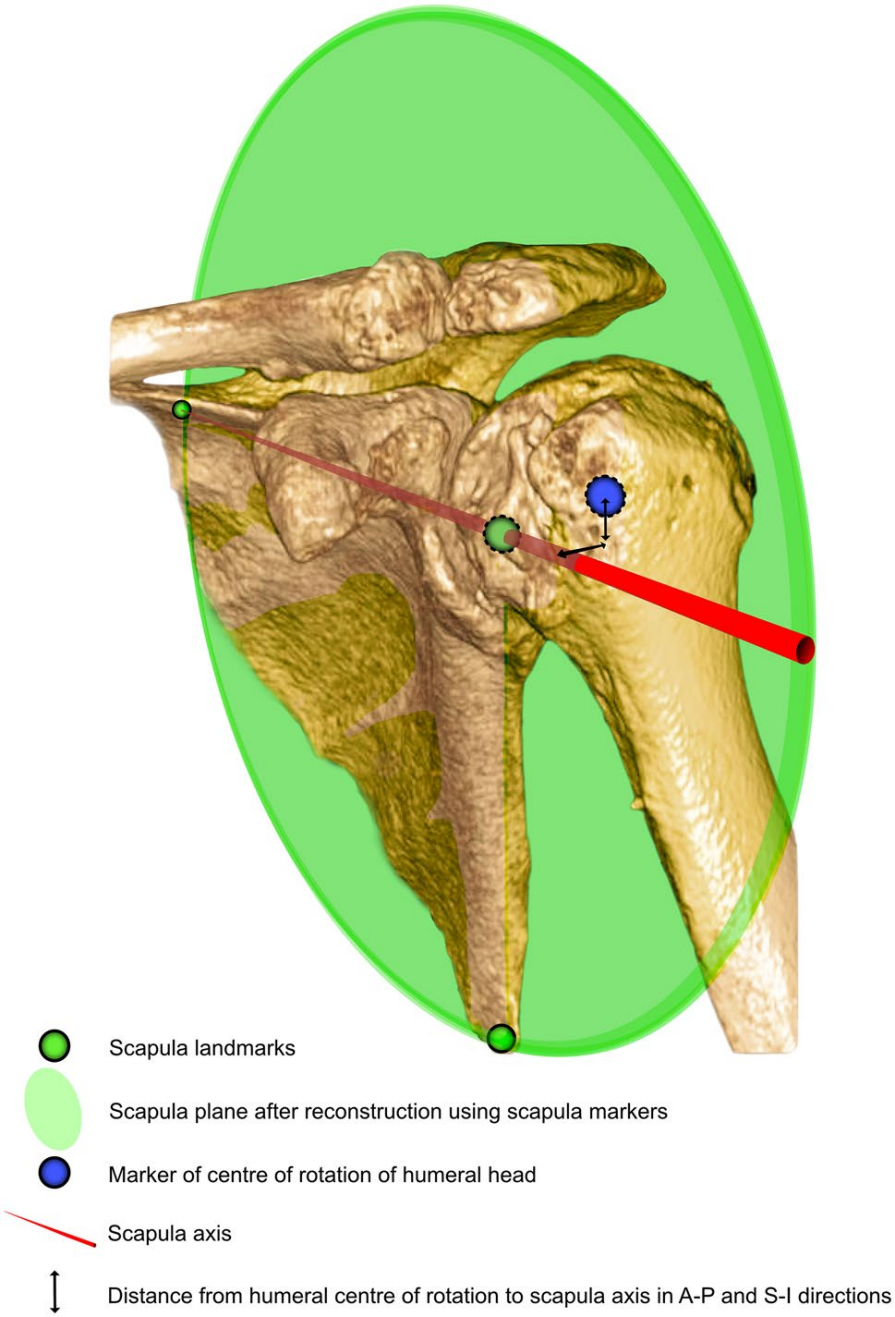
Illustration of the scapular plane and its landmarks (trigonum, glenoid center, and inferior angle of the scapula) on a three–dimensional reconstruction of a shoulder CT. The scapular axis, in reference to which alignment in the anteroposterior and superoinferior directions was measured, is the red line passing from the trigonum through the glenoid center [15]

**Fig. 2:**
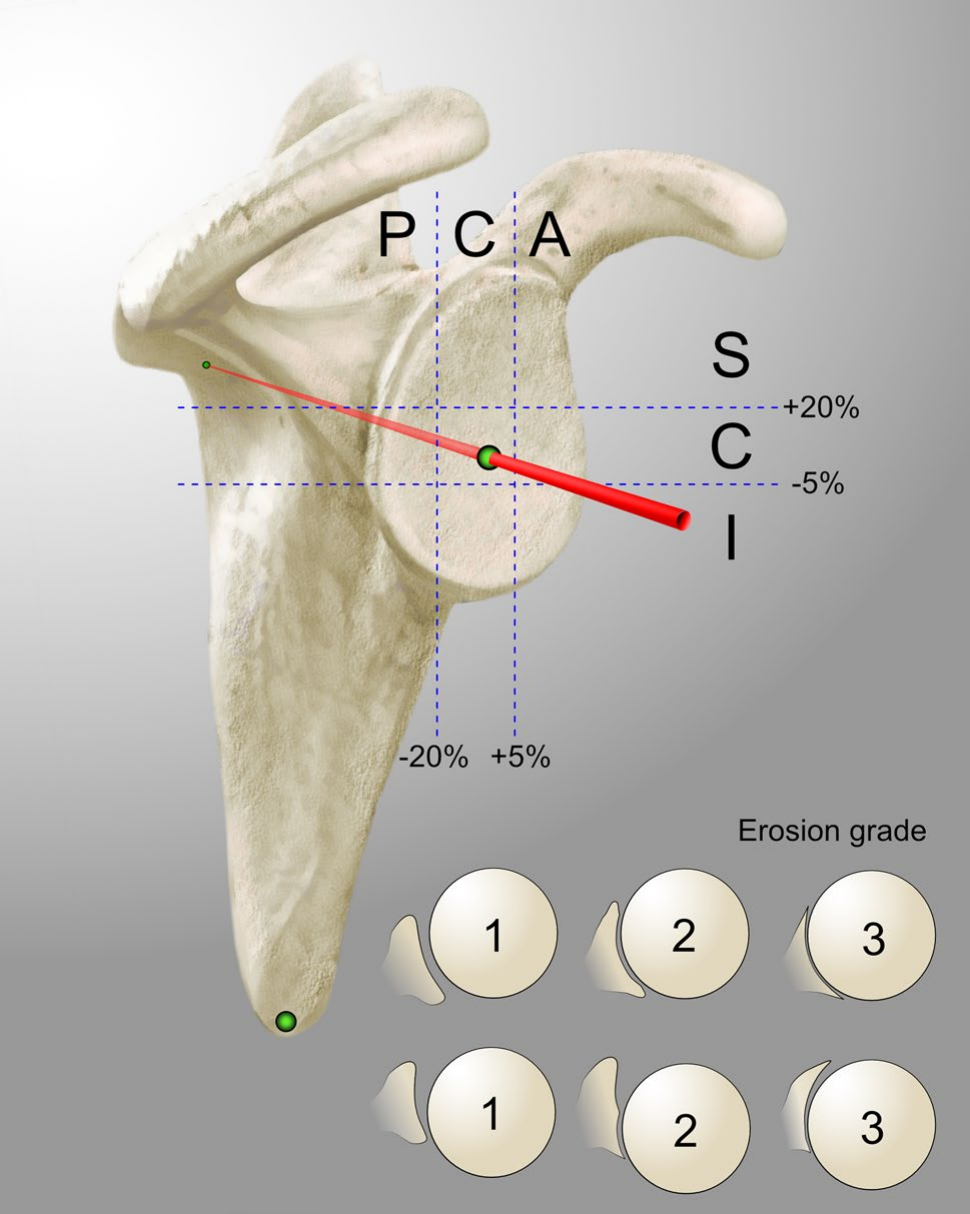
Schematic illustration of the three–dimensional classification for degenerative arthritis of the shoulder. The humeroscapular alignment was described in terms of alignment of the center of rotation of the humeral head in relation to the scapula axis in the anteroposterior direction (posterior [P]/central [C]/anterior [A]) and the superoinferior direction (superior [S]/central [C]/inferior [I]), for a total of nine different combinations. Erosion was graded from 1 to 3, where 1 = no significant bony erosion, 2 = focal erosion forming a crater or biconcavity of the glenoid (central or eccentric), and 3 = severe glenoid erosion involving the entire glenoid surface in any single plane (central or eccentric) [15]

**Fig. 3:**
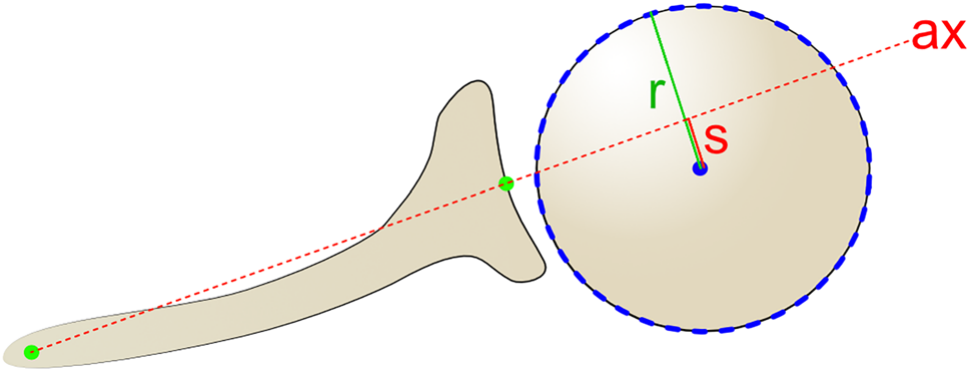
Method for determining the percentage of subluxation, *s*, of the humeral head center from the scapular axis, ax, relative to the radius of the humeral head, *r* [15]

A clinical image viewing software capable of 3D reconstruction (IDS7 Workstation Version 22.2; Sectra) was used to classify CT images according to the previously described 3D classification for DAS [15]. First, the scapular plane was reconstructed in 3D using two-dimensional orthogonal planes (axial, coronal, and sagittal): The glenoid center, trigonum and inferior angle of the scapula were aligned in one plane (Fig. 1). Following this, anteroposterior alignment was classified as posterior, centered or anterior; superoinferior alignment was classified as superior, centered or inferior and combined with an erosion grade (1–3) (Fig. 2) [15]. Subluxation of the humeral head center from the scapular axis (line passing from trigonum scapulae through glenoid center) was assessed relative to the radius of the humeral head (Fig. 3):

$$\% \;{\text{of subluxation}}\; = \;\frac{{{\text{distance of center of humeral head from scapula axis}} }}{{{\text{radius of the humeral head}} }} \times 100$$

**Fig. 4:**
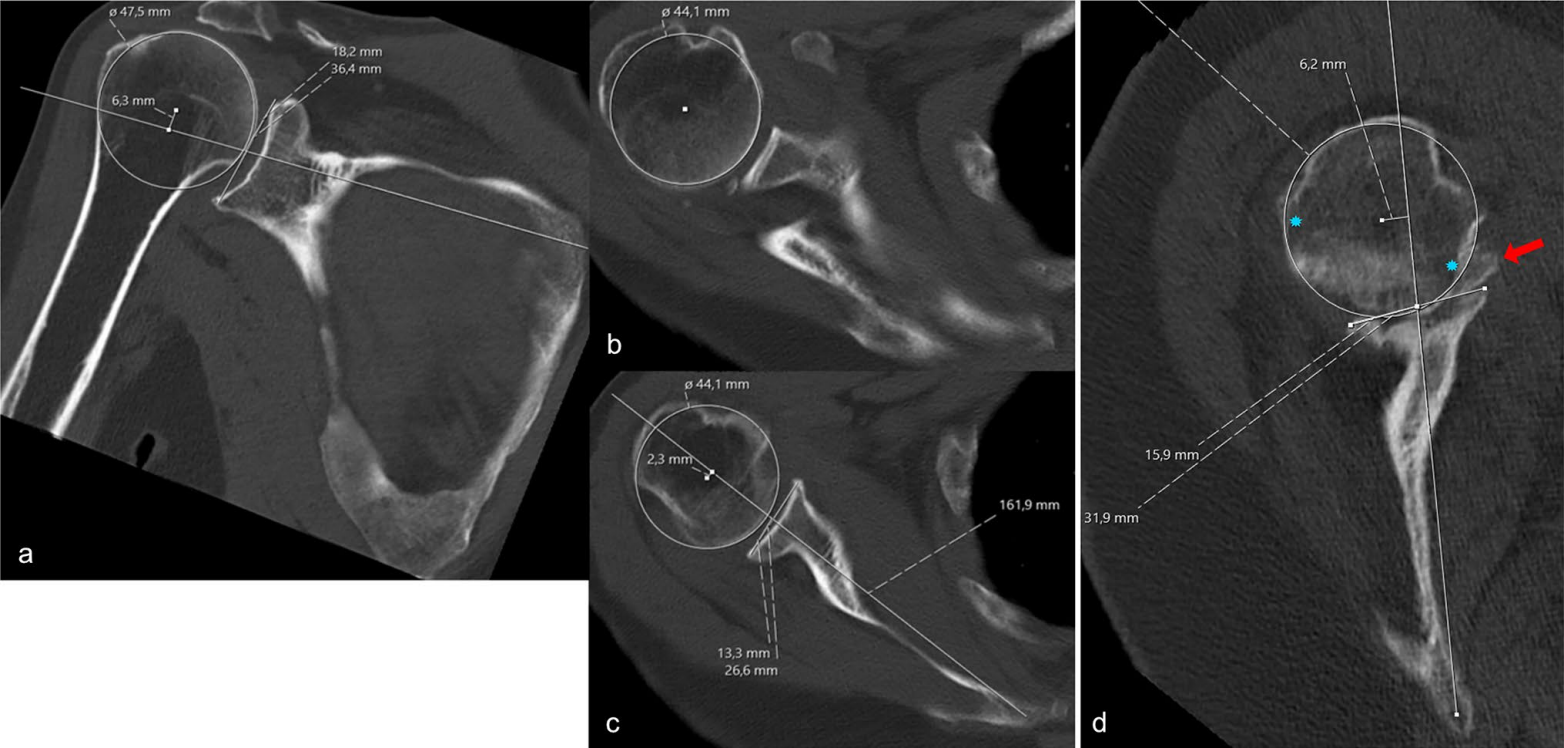
Method for measurement of humeroscapular alignment on **A** coronal plane and **B** and **C** cross–sectional plane computed tomography scans, after reconstruction of the scapular plane in three dimensions. (B) The center of rotation of the humeral head is determined at the widest cross–section of the head and (**C**) is then translated down to the level of the scapular axis for measurement of subluxation. (**D**) When higher–grade erosion with partial humeral head collapse and osteophytes are present, measurement is more challenging. Osteophytes (red arrow), whether on the humeral or glenoid side, are disregarded. The center of rotation is determined using the intact outer margins of the humeral joint surface (blue stars) as landmarks [15]

Centered alignment was defined as: Between 20% posterior and 5% anterior subluxation in the anteroposterior direction; between 5% inferior and 20% superior subluxation in the superoinferior direction, as previously described [15]. Additionally, alignment could be described as extra-posterior if posterior subluxation was > 60% of the humeral head *radius* (> 80% of the *diameter*), or extra-superior if static acetabularization was present [15]. Where alignment seemed to be obvious, observers were not required to perform measurements. In borderline cases the scapula axis, humeral circumference with center point, radius and subluxation of the center of the humeral head from the scapula axis was determined to quantify the classification (Fig. 4).

After a training seminar of 2 h, four orthopedic residents with experience in shoulder surgery (none of which were involved in measurements for the primary study) independently classified all 100 CT scans. An instructional handout for the 3D reconstruction and classification method, including a schematic representation of the 3D classification (Fig. 2) was provided. Four weeks after the first analysis the same four observers classified the 100 CT scans for a second time. Observers were blinded to the results of their previous attempt. The time needed to apply the new classification was measured for each observer. To simulate time available in clinical practice, observers were instructed to aim to take < 5 min per CT.

The humeroscapular alignment of all 100 shoulder CTs had previously been precisely measured and classified for the primary study by BDK and MH, with almost perfect interobserver agreement for alignment measurements [15]. Erosion was classified in consensus between the two observers. These existing classification values (shown in Table 2) were taken as the gold-standard against which the present values of the Observers were compared for validation.

**Table 2:** Case distribution of 100 CTs analyzed for the present study according to the 3-dimensional classification, based on measurements from the primary study [15]

	EG	Posterior	Central	Anterior
Superior	1 2 3	6 12 1	16 10 5	– 3 –
Central	1 2 3	– 10 13	4 10 3	– 2 –
Inferior	1 2 3	– 3 1	– 1 –	– – –

*EG* erosion grade

Additionally, 15 shoulders were described as having extra–superior and 4 as extra–posterior alignment

### Statistical analysis

Statistical analysis was performed using SPSS Version 29.0 (IBM-SPSS, New York, USA) software. Classification values were recoded and entered as string variables with three ordinal categories. Intraobserver reliability, interobserver reliability and validity (compared to existing gold-standard values) were calculated using Cohen’s weighted κ with linear weighting. Mean averages of comparisons of both attempts from each observer (four comparisons for each individual intraobserver comparison) were calculated for all six individual interobserver comparisons between the four observers. A 95% confidence interval (CI) was determined for each value. Cohen’s categorization of agreement (≤ 0 indicating no agreement, 0.01–0.20 none to slight, 0.21–0.40 fair, 0.41– 0.60 moderate, 0.61–0.80 substantial and 0.81–1.00 almost perfect agreement) was taken for interpretation of κ values [21]. Tests were two-sided, with a significance level of 0.05.

## Results

The intraobserver reliability of the 3D classification showed substantial agreement with a Cohen’s weighted *κ* of 0.71 (CI 0.63–0.79).

Interobserver agreement was moderate overall, ranging from fair to substantial (Table 3). When assessing agreement for biplanar alignment only (disregarding erosion grade), interobserver agreement improved in each comparison, ranging from moderate to substantial, although differences were not statistically significant (Table 3). Interobserver agreement did not change substantially when the additional descriptors of extra-posterior and extra-superior were included (*κ* = 0.44 [CI 0.33–0.55]).

**Table 3:** Interobserver agreement using Cohen’s weighted kappa for biplanar alignment with erosion values (upper half) and biplanar alignment only (lower half)

Alignment with erosion	Observer 4 (Attempts 1 and 2)	Observer 3 (Attempts 1 and 2)	Observer 2 (Attempts 1 and 2)
Observer 1 (Attempts 1 and 2)	0.37 (0.26–0.48)	0.67 (0.61–0.73)	0.46 (0.34–0.57)
Observer 2 (Attempts 1 and 2)	0.40 (0.27–0.54)	0.49 (0.38–0.61)	
Observer 3 (Attempts 1 and 2)	0.38 (0.27–0.50)		
Overall mean average	**0.46 (0.36–0.57)**		

Alignment only	Observer 4 (Attempts 1 and 2)	Observer 3 (Attempts 1 and 2)	Observer 2 (Attempts 1 and 2)
Observer 1 (Attempts 1 and 2)	0.41 (0.30–0.54)	0.71 (0.64–0.78)	0.55 (0.42–0.68)
Observer 2 (Attempts 1 and 2)	0.47 (0.34–0.60)	0.61 (0.48–0.73)	
Observer 3 (Attempts 1 and 2)	0.46 (0.33–0.59)		
Overall mean average	**0.55 (0.42–0.65)**		

The mean average agreement for comparisons of both attempts from each observer with both attempts from all other observers is displayed alongside a 95% confidence interval. The overall mean for each analysis is highlighted in bold

The validity analysis, comparing the observers’ classifications to the gold-standard values, showed moderate agreement (Table 4). As observed in the interobserver analysis, when the additional descriptors of extra-posterior and extra-superior were included the agreement for validity did not change substantially (*κ* = 0.45 [CI 0.33–0.56]). When analyzing the quantifiable aspect (biplanar alignment) only, *κ* increased to 0.53 (CI 0.41–0.65).

**Table 4:** Results of the validity analysis showing the mean average of both attempts from each observer with the gold standard values

	Agreement with gold standard
Observer 1	0.49 (0.37–0.61)
Observer 2	0.52 (0.41–0.63)
Observer 3	0.53 (0.41–0.64)
Observer 4	0.37 (0.25–0.48)
Total average	0.48 (0.36–0.59)

Cohen’s weighted kappa values are displayed with a 95% confidence interval

The mean average for time taken for classification was 2 min and 47 s (range 45 s to 4 min 1 s).

### Key findings

The most important finding of this study was that interobserver agreement (reproducibility) for the 3D classification for DAS was moderate (*κ* = 0.46). Despite being more comprehensive than these, agreement for the 3D classification is at least comparable to that reported for previous monoplanar (two-dimensional) classifications for CTA or OA of the shoulder (Table 1) [16–20]. This is likely to be due to the quantifiable nature of the alignment aspect of this classification, which allows for clear cut-off values. Furthermore, observers were able to apply the classification in < 5 min, which reflects time available in clinical practice.

### Interpretation

The most recently published study to compare intra- and interobserver agreement for the modified Walch classification (modifications by Bercik and Iannotti) is the most comparable to the present study, as it also employed a 3D image viewing software for analysis of CT images [1, 16, 22]. This study also found moderate inter- and moderate to substantial intraobserver agreement, comparable to the results of the present study [16]. The authors did not find an improvement in agreement when considering the alignment groups only (without erosion subgrouping). Although there was a trend to improved agreement when disregarding erosion grading, this was also not statistically significant in the present analysis.

An equally recent study investigating the original Walch classification found substantial intraobserver but only fair interobserver agreement for both CT and MRI images [18]. Shukla et al. found substantial intra- and moderate interobserver agreement of the modified Walch classification on both X-ray and CT [17]. They suggest the use of automated computer-based analysis of CT scans to improve the reproducibility of the modified Walch classification further. Whilst the modifications to the Walch classification have improved interobserver agreement somewhat, it remains a two-dimensional classification with subjective intergroup cut-offs [1, 22]. The presently investigated 3D classification is quantifiable with precise cut-off values and may therefore be better suited to such an automated computer-based software algorithm. Being 3D, it is more aligned with current developments in imaging software, preoperative planning and instrumentation than previous two-dimensional classification systems [23–25]. Modern 3D planning software already comprise algorithms which calculate the anteroposterior subluxation and these merely need to be modified to include superoinferior alignment [24, 26].

Established classification systems for CTA are all based on AP X-ray images and show variable levels of intra- and interobserver agreement [19, 20]. Whilst the Visotsky-Seebauer and Hamada classifications showed substantial to almost perfect intraobserver agreement with moderate interobserver agreement, the Favard classification was found to have moderate intra- and none to slight interobserver agreement [19, 20]. Despite its higher complexity, the presently investigated 3D classification shows comparable intra- and interobserver agreement to those established CTA classifications with better agreement (Visotsky-Seebauer and Hamada). These seminal classifications helped lead to a greater understanding of the disease process, but are now based on outdated technology. To further differentialize diagnosis, treatment and outcomes for DAS for clinical application and in research, use of the 3D classification should be considered.

A validation analysis is new to the present study of the 3D classification for DAS. This can be performed as the alignment of the classification is quantifiable and, therefore, *correct* classification is possible. The erosion subgrouping aspect is, as in the previous classifications subject to interpretation. Statistically significant moderate agreement was found in this validity analysis, rendering it valid for the classification of DAS. Validity agreement was moderate both for alignment alone and for alignment with erosion grade. As agreement for alignment was only moderate when detailed measurements are not performed ubiquitously (simulation of clinical practice), this has potential to be improved using automated software algorithms in the future.

### Limitations

The validity analysis of this study depends on gold-standard values, of which only the alignment aspect is quantifiable. Despite being determined by two orthopedic surgeons in consensus the erosion aspect is, as in previous classifications, subject to some interpretation. The analysis, therefore, gives a breakdown of these aspects. As the 3D classification and method to reconstruct the scapular plane was new to the four observers, the training provided may not have been sufficient to get the best results possible. However, this reflects clinical practice as future users of this classification will not receive training beyond reading the published information available. As all observers in the present study were orthopedic residents, a difference in performance for varying levels of expertise could not be investigated.

### Generalizability

As a wide variety of classification types were examined in a large sample of patients with DAS by four independent observers, the results of this study can be applied widely to classification of DAS. Availability of an image viewer with the ability of 3D planar reconstruction is, however, a prerequisite for reliable use of this classification.

## Conclusion

The 3D classification for DAS is valid. Despite being more comprehensive, the classification shows intra- and interobserver agreement comparable to previously established classifications for DAS. Being quantifiable, this has potential for improvement with automated algorithm-based software analysis in the future. The classification can be applied in under 5 min and thus can be used in clinical practice.

## Data Availability

The datasets generated and analyzed during the current study are not publicly available due to institutional data protection agreements but are available from the corresponding author on reasonable request.
