# Correction to: Validation of a novel 3‑dimensional classification for degenerative arthritis of the shoulder

**DOI:** 10.1007/s00402-023-04995-8

**Published:** 2023-08-02

**Authors:** Benjamin D. Kleim, Sebastian Lappen, Pavel Kadantsev, Hannes Degenhardt, Lorenz Fritsch, Sebastian Siebenlist, Maximilian Hinz

**Affiliations:** Department of Sports Orthopaedics, Technical University of Munich, Ismaninger Str 22, 81675 Munich, Germany

**Correction to:** **Archives of Orthopaedic and Trauma Surgery** 10.1007/s00402-023-04890-2

The original version of this article unfortunately contained a mistake. The first names and last names of the authors have been swapped.

The author names (surname, given name(s)) should be:

Kleim, Benjamin D.

Lappen, Sebastian

Kadantsev, Pavel

Degenhardt, Hannes

Fritsch, Lorenz

Siebenlist, Sebastian

Hinz, Maximilian

The original article has been corrected.

